# Childhood maltreatment and subsequent depressive symptoms: a prospective study of the sequential mediating role of self-esteem and internalizing/externalizing problems

**DOI:** 10.1186/s12888-023-04654-7

**Published:** 2023-03-20

**Authors:** Wenyan Li, Wenjian Lai, Lan Guo, Wanxin Wang, Xiuwen Li, Liwan Zhu, Jingman Shi, Kayla M. Teopiz, Roger S. McIntyre, Ciyong Lu

**Affiliations:** 1 Department of Medical Statistics and Epidemiology, School of Public Health, Sun Yat-Sen University, 74 Zhongshan Rd 2, Guangzhou, 510080 China; 2 Mood Disorders Psychopharmacology Unit, University Health Network, Toronto, ON Canada; 3 Department of Pharmacology, University of Toronto, Toronto, ON Canada; 4 Department of Psychiatry, University of Toronto, Toronto, ON Canada; 5 Institute of Medical Science, University of Toronto, Toronto, ON Canada

**Keywords:** Depressive symptoms, Childhood maltreatment, Self-esteem, Internalizing and externalizing problems, Sex differences

## Abstract

**Background:**

Depression among adolescents is a seriously disabling public health problem with an extremely high prevalence. Identifying risk factors of depression at an early stage is important to reduce the disease burden. Childhood maltreatment (CM) is one of the major risk factors for depression. The key mediating processes that how CM affects the development of depression, however, still need further clarification. The present study tested the mediating effect of self-esteem, internalizing problems, and externalizing problems between CM and depressive symptoms. Potential sex differences in the foregoing associations were also explored.

**Methods:**

A three-wave longitudinal study was carried out among 1,957 middle and high school students from 69 classes in 10 public schools in the Guangdong province of China. Data collection started when students were in grades 7 and 10 (median age: 13.0, range: 11–18) between January and April 2019, and the students were followed up once a year thereafter. Self-reported CM, depressive symptoms, self-esteem, internalizing and externalizing problems, and other demographics were collected. The multiple serial mediation analysis was conducted.

**Results:**

We found that CM was positively related to subsequent internalizing and externalizing problems, as well as depressive symptoms, while self-esteem was negatively related to depressive symptoms. Serial mediation analysis indicated that self-esteem (mediator 1) and internalizing problems (mediator 2) sequentially mediated the path from CM to depressive symptoms in the overall and male population. Moreover, with externalizing problems as mediator 2, self-esteem (mediator 1) acted as a partial mediator in the association between CM and depressive symptoms in males, whereas externalizing problems played a complete mediating role in females.

**Conclusion:**

Findings revealed that self-esteem and internalizing problems sequentially mediated the influence of CM on depressive symptoms whereas externalizing problems played an independent mediating role. In addition, sex differences need to be taken into consideration when designing prevention and intervention strategies, given the different psychosocial processes between boys and girls.

**Supplementary Information:**

The online version contains supplementary material available at 10.1186/s12888-023-04654-7.

## Background

Depressive symptoms are increasingly recognized as a serious public health problem worldwide [1]. Adolescence is an important period of growth and development as well as a key risk period when the occurrence of depressive symptoms rises sharply [2]. Depressive symptoms are the most common cause of years lived with disability among adolescents, according to the Global Burden of Disease study [3]. Furthermore, during adolescence, sex differences emerge and widen dramatically in regard to emotional disorders, however, these differences do not appear in childhood [4]. Depressive symptoms are two to three times more prevalent in girls compared to boys during adolescence, and girls are also affected more seriously by it [5, 6]. However, the etiology of depression and the specific factors contributing to sex differences are still unclear [7]. Identifying potential risk factors for depressive symptoms may help develop effective preventive strategies [8]. Among the numerous risk factors for depressive symptoms, childhood maltreatment (CM) is one of the most important risk factors exerting profound influences on the development of depression [9, 10].

Childhood maltreatment refers to the emotional abuse/neglect, physical abuse/neglect, as well as sexual abuse of children under the age of 18 years [11]. Childhood maltreatment has been consistently considered a robust predictor of depressive symptoms [9, 10]. According to the hopelessness theory, negative life events (e.g., CM) can serve as distal risk factors, and increase the risk of hopelessness (i.e., proximate risk factors), ultimately leading to the occurrence of depression [12]. Furthermore, attachment theory maintains that children who experience CM are likely to develop negative representations of themselves, subsequently developing insecure attachment styles [13, 14]. In addition, attachment insecurity can lead to more problems in interpersonal relationships and social withdrawal, which is linked to depressive symptoms [15, 16]. Thus, CM is associated with depressive symptoms during adolescence and increases the risk of adverse psychological health consequences. A study of adolescents in China revealed that CM was positively related to depressive symptoms [17]. Furthermore, a meta-analysis of 192 studies concluded that the diagnosis and scores of depression were positively correlated with each type of CM [9]. Recently, a separate meta-analysis reported a dose-response relationship between multiple forms of maltreatment and the severity of depression [10]. Despite compelling evidence about the detrimental effect of CM on depressive symptoms, questions about mediating factors contributing to the foregoing association remain largely unanswered [18]. Thus, it is necessary to illuminate important mediating mechanisms that could serve as potential targets for intervention.

Self-esteem, as well as emotional and behavioral problems, are important indicators of mental health. Self-esteem is the sense in which people think they are worthwhile as human beings with a significant effect on promoting adaptive capacity [19]. Adolescence is a critical developmental period of self-esteem formation while self-esteem appears to be a significant contributor to mental health throughout adolescence [20]. During adolescence, a vulnerable stage, CM has the potential to undermine confidence and self-esteem, with a greater probability of leading to long-term health effects [21]. High self-esteem may promote more prominent happiness and goal achievement, while low self-esteem is associated with ambivalent feelings or avoidance, then possibly leads to depressive symptoms [19]. Previous researchers have explored the relationship between self-esteem and depressive symptoms, consistently finding that low self-esteem is a significant predictor of future depressive symptoms [22, 23]. Moreover, it has been reported that experiencing maltreatment in early life inversely affected the development of self-respect and dignity [24]. Recent longitudinal research in many nations, including the United States and China, have shown that the relationship between CM and depressive symptoms was mediated by self-esteem in adolescents [18, 25]. However, a cross-sectional study in Japan did not find a significant mediating effect of self-esteem on the relationship between CM and depressive symptoms [26].

Emotional and behavioral problems in adolescents can be divided into internalizing problems (emotional disorders like depression, fearfulness, and anxiety) and externalizing problems (disruptive behaviors like aggression, defiance, and hyperactivity) [27]. Exposure to CM may give rise to internalizing and externalizing problems [28]. Our previous findings showed that internalizing problems mediated the association of CM and depressive symptoms [29]. Nevertheless, longitudinal data suggested that CM predicted childhood internalizing and externalizing problems that culminated in the development of depression in emerging adulthood, but the mediating effect of the internalizing problems was not statistically significant [30]. Thus, there is a need for more extensive and in-depth research to explore the role of self-esteem, internalizing problems, and externalizing problems in the relationship between CM and depressive symptoms.

Furthermore, low self-esteem has longitudinal associations with internalizing and externalizing problems [31, 32]. A study assessing adolescents aged 13 ~ 18 years reported that adolescents with high self-esteem were less likely to have internalizing and externalizing problems after three years [31]. Similarly, a separate outpatient psychiatric clinic study reported that adolescents with low self-esteem had more severe internalizing symptoms at baseline, and had higher internalizing symptoms at posttreatment [32]. Recently, a study conducted on adolescents revealed that the association between childhood adversity and internalizing and externalizing behavior problems was mediated by self-esteem [33]. The foregoing findings provide support for the hypothesis that self-esteem and internalizing/externalizing problems may sequentially mediate the association between CM and depressive symptoms.

It is well established that there is a sex difference in depressive symptoms, but the exact cause has not been determined. Observed sex differences in persons affected by depressive symptoms may be attributed to biological variances or social expectations and experiences [34]. One study reported that males generally exhibit a higher level of self-esteem than females [35], while externalizing disorders were observed more frequently in males and internalizing disorders in females [36]. However, relatively few studies investigated potential mediational pathways underlying observed sex differences in the association between CM and depressive symptoms [18, 25, 29]. Taken together, we hypothesized that there might be sex differences in the effects of CM on depressive symptoms through self-esteem and internalizing/externalizing problems.

**Fig. 1:**
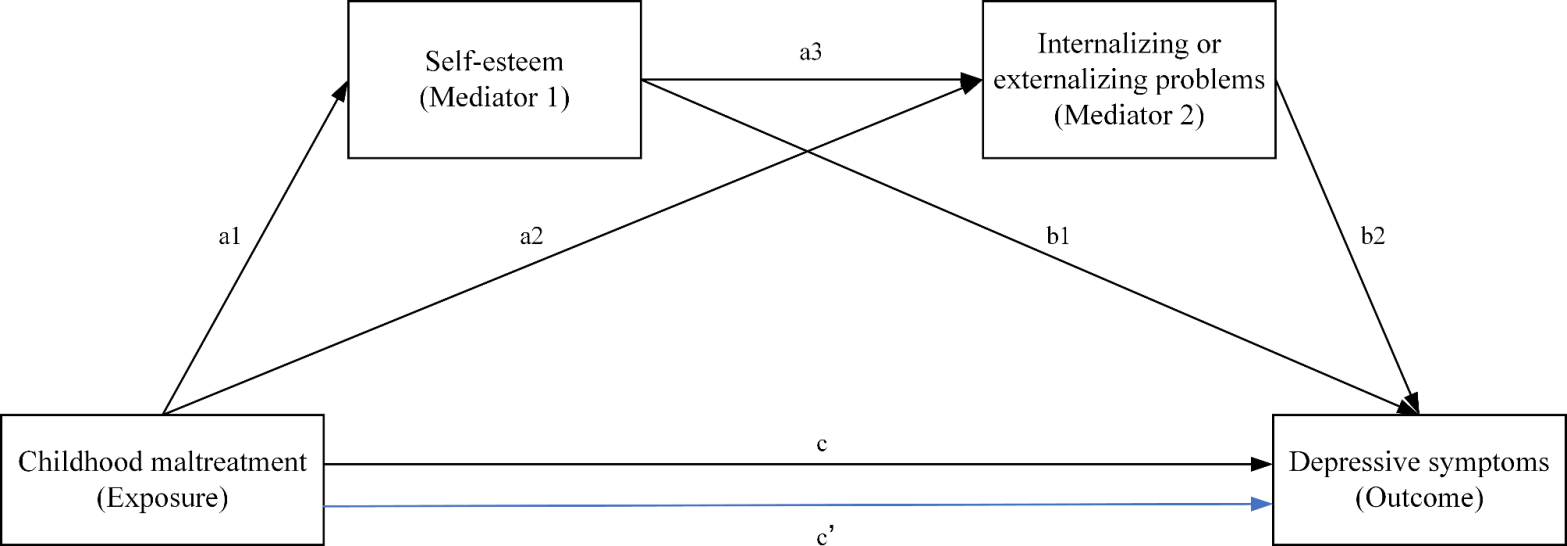
Conceptual multiple serial mediation model Hypothesized indirect effect of CM at wave 1 (exposure variable) on the severity of depressive symptoms at wave 3 (outcome variable) via self-esteem at wave 1 (mediator 1) and internalizing/externalizing problems at wave 2 (mediator 2). Path c’ and c: total and direct effect of exposure on the outcome; paths a1 and a2: effect of exposure on mediator 1 and mediator 2; path a3: effect of mediator 1 on mediator 2; path b1 and b2: mediator 1 and mediator 2 on the outcome

The current study aimed to test the interrelationship between CM, self-esteem, internalizing/externalizing problems, and depressive symptoms in adolescents. A second aim of the study herein was to explore whether self-esteem and internalizing/externalizing problems play serial-mediation roles in the relationship between CM and depressive symptoms. The theoretical hypothesis model was established as shown in Fig. 1. We hypothesized that: (1) low self-esteem would play a mediating role in the relationship between CM and depressive symptoms; (2) internalizing/externalizing problems would play a mediating role in the relationship between CM and depressive symptoms; (3) self-esteem and internalizing/externalizing problems serially mediated the relationship between CM and depressive symptoms; and (4) there were sex differences in the above associations.

## Methods

### Study design and participants

Data was obtained from the Longitudinal Study of Adolescents’ Mental and Behavioral Well-being Research (LSAMBR) in Guangzhou, China (Registration No. ChiCTR1900022032), which has been described in detail elsewhere [37]. A total of 1957 seventh- and tenth-grade students from 69 classes in 10 public schools were enrolled in the LSAMBR at baseline (wave 1; January to April 2019; response rate: 99.0%). The follow-up assessments were conducted at 12 months (wave 2; n = 1836; retention rate: 93.8%) and 24 months (wave 3; n = 1791; retention rate: 91.5%) after the baseline assessment, following the same procedures. Attrition was mainly due to students’ transfer out of school and absenteeism on the day of the data collection. We measured both the predictor variable (CM) and the mediator variable (self-esteem) at wave 1, assessed the mediator variable (internalizing and externalizing problems) at wave 1 and wave 2, and measured the dependent variable (depressive symptoms) at wave 1 and wave 3.

Before the study commenced, a note was sent to the students and their parents explaining the purpose of the study, the voluntary nature of joining, and the confidentiality of the responses. Written informed consent was obtained from all participating students as well as one of their parents. Participants were invited from all selected classes, and those who could not give informed consent were excluded from the study. Given the sensitivity of some questions, all participants were convened in their classrooms simultaneously, and the anonymous self-reported questionnaires were completed independently without the presence of any teachers or school administrators. Ethical approval was granted by the Sun Yat-sen University, School of Public Health Institutional Review Board (Ethics Number: L2017060).

### Measures

#### Childhood maltreatment

Childhood maltreatment was assessed using the Childhood Trauma Questionnaire-Short Form (CTQ-SF) [38], a short version of the Childhood Trauma Questionnaire developed by Bernstein et al. [39, 40]. The CTQ-SF is a 25-item questionnaire consisting of five subscales, including physical neglect, emotional neglect, emotional abuse, physical abuse, and sexual abuse, each containing five items. Participants were required to rate the frequency of each maltreatment phenomenon occurring in childhood on a 5-point Likert scale (1 = never, 2 = rarely, 3 = sometimes, 4 = often, and 5 = very often). Each type of maltreatment was scored from 5 to 25. The sum of all 25 item scores resulted in a total score, with higher scores indicating more frequent exposure to CM. Total CTQ score was often used as a continuous variable based on prior research suggesting CM exhibits a dose-dependent association between multiple forms of abuse and neglect on mental disorders [10, 41]. The Chinese version of the CTQ-SF has good reliability and validity in assessing children and adolescents in China [42]. Scale reliability for the present sample was calculated via the McDonald’s omega coefficient and a value greater than 0.70 was considered acceptable [43]. The McDonald’s omega coefficient in this study was 0.80.

#### Self-esteem

Self-esteem was assessed using the Rosenberg Self-Esteem Scale (RSES) originally developed by Rosenberg, a widely used 10-item measure of global self-esteem including five positive items (e.g., “I feel that I am a person of worth, at least on an equal basis with others”), and five negative items (e.g., “I feel I do not have much to be proud of”) [44]. Each item is rated on a 4-point Likert scale ranging from 1 (strongly agree) to 4 (strongly disagree). Positive items were reversely scored. The total score was the sum of all items (ranging from 10 to 40 points), with higher scores indicating greater self-esteem. The Chinese version of the RSES been validated with good reliability and validity and is widely used in studies on Chinese adolescents [45, 46]. The McDonald’s omega coefficient of RSES was 0.82 in this study.

#### Internalizing and externalizing problems

Adolescents’ internalizing and externalizing problems were assessed using the Strengths and Difficulties Questionnaire (SDQ) [47]. This scale consists of 25 questions and 5 dimensions of mental health problems (emotional symptoms, peer problems, conduct problems, hyperactivity-inattention, and prosocial behavior). It is a 3-point Likert scale, with scores ranging from 0 (not true) to 2 points (certainly true). According to the recommendations of Goodman et al. [48], we divided SDQ into two broader subscales: internalizing problems (peer and emotional problems) and externalizing problems (conduct problems and hyperactivity-inattention) subscale, with higher scores indicating higher levels of difficult behaviors. In this study, the prosocial behavior domain was not used, considering its conceptual and statistical independence from internalizing and externalizing problems [49, 50]. These scales have shown good internal consistency and construct validity [47, 48, 51]. The Chinese version of SDQ also showed acceptable reliability and validity [51]. This scale had a McDonald’s omega coefficient of 0.76 and 0.72 for wave 1 and wave 2, respectively.

#### Depressive symptoms

Depressive symptoms were assessed using the 20-item Center for Epidemiologic Studies Depression Scale (CESD) [52]. This instrument evaluates the frequency of common depressive symptoms that occurred in the last week, with each item rating on a 4-point scale (0 = rarely, 1 = 1–2 days per week, 2 = 3–4 days per week, and 3 = 5–7 days per week). The total score ranged from 0 to 60, with higher scores indicating more severe depressive symptoms. The CESD showed good reliability in our study (wave 1, omega coefficient = 0.90; wave 3, omega coefficient = 0.91).

#### Potential covariates

The following variables will be assessed as potential covariates. In wave 1, participants’ self-reported characteristics were collected, including age, sex, household socioeconomic status (HSS), and living arrangement. Household socioeconomic status was assessed based on the question “What is the financial status of your family?”. The five response options were aggregated into three categories: excellent (very good), good, and fair/poor (middle, poor, or very poor). The living arrangements were categorized into “living with both parents”, “living with a single parent” and “living with others”. Relationships with classmates/teachers were assessed with the question “How would you describe your relations with your classmates/teachers?” (1 = good, 2 = average, and 3 = poor). Cigarette smoking and alcohol consumption were assessed by the following questions: “Have you ever smoked a whole cigarette?” and “Have you ever drunk beer, wine, or liquor?” (1 = yes, 2 = no).

#### Data analysis

Descriptive information and correlation matrix were analyzed using IBM SPSS Statistics (V.25, IBM Corporation, New York, USA). First, descriptive analyses of the baseline characteristics were made, stratified by sex. Categorical variables were described by numbers (percentages), and nonnormally distributed continuous variables were described by median [interquartile range]. The chi-square test or Wilcoxon-Mann-Whitney test was used to compare the differences. Second, the Spearman rank-order correlation analysis was used for the assessment of correlation between CM, self-esteem, internalizing/externalizing problems, and depressive symptoms. Third, the serial mediation analysis was conducted in Hayes [53] PROCESS macro, version 3.3, model 6. We hypothesized that the relationships between CM (predictor variable at wave 1) and depressive symptoms (dependent variable at wave 3) were sequentially mediated by self-esteem (mediator 1 at wave 1) and internalizing/externalizing problems (mediator 2 at wave 2). The multiple serial mediations assumed that multiple mediators along specific directions form a causal chain. For all serial mediation models, we estimated one direct effect, three specific indirect effects, total indirect, and total effects of the predictor variable on the dependent variable (Fig. 1). The total effects (path c’) comprised a direct effect pathway (path c) of CM on depressive symptoms and a total indirect pathway (mediated: path a1 × b1 + path a2 × b2 + path a1 × a3 × b2) of CM on depressive symptoms through self-esteem and internalizing/externalizing problems.

The 95% confidence interval (CI) was obtained with 5000 bootstraps resamples, and 95% CI that did not overlap zero indicated significant indirect effects. Effect sizes were expressed as non-standardized estimates. Serial multiple mediation analysis was performed in the entire study population and then repeated with stratifying sex. Age, household socioeconomic status, living arrangement, classmate relations, relationships with teachers, smoking, drinking, depressive symptoms, internalizing/externalizing problems at wave 1, and sex (overall model only) were included as covariates in all mediation models.

## Results

### Demographic characteristics

Baseline demographic characteristics by sex are summarized in Table 1. Of the 1957 participants, 994 (50.8%) were male. The median for age of the students was 13.0 years (age range, 11 ~ 18 years). Males were more likely to be ever smokers and alcohol drinkers, live with others, and have poor relationships with teachers (*p* < 0.05). The distribution medians are significantly different in the age, total RSES scores, baseline CESD scores, and internalizing problems of the two groups (*p* < 0.05). No sex difference in HSS, classmate relations, total CTQ scores, and baseline externalizing problems was found.

**Table 1:** Baseline characteristics of 1,957 participants stratified by sex

Variables^&^	Total sample (*n* = 1957)	Males (*n* = 994, 50.8%)	Females (*n* = 963, 49.2%)	*p* ^*^
Age (year)	13.0 [13.0, 15.0]	13.0 [13.0, 15.0]	13.0 [12.0, 15.0]	0.025
HSS				0.058
Excellent	191 (9.8)	104 (10.5)	87 (9.1)	
Good	821 (42.1)	391 (39.5)	430 (44.7)	
Fair or poor	939 (48.1)	495 (50.0)	444 (46.2)	
Missing data	6	NA	NA	
Living arrangement				0.047
Living with both parents	1594 (81.6)	805 (81.2)	789 (82.0)	
Living with a single parent	192 (9.8)	88 (8.9)	104 (10.8)	
Living with others	167 (8.6)	98 (9.9)	69 (7.2)	
Missing data	4	NA	NA	
Classmate relations				0.522
Good	1663 (85.2)	838 (84.6)	825 (85.9)	
Average	254 (13.0)	137 (13.8)	117 (12.2)	
Poor	34 (1.7)	16 (1.6)	18 (1.9)	
Missing data	6	NA	NA	
Relationship with teachers				0.002
Good	1605 (82.8)	792 (80.4)	813 (85.2)	
Average	307 (15.8)	172 (17.5)	135 (14.2)	
Poor	27 (1.4)	21 (2.1)	6 (0.6)	
Missing data	18	NA	NA	
Ever smoking a cigarette				0.010
Yes	28 (1.4)	21 (2.1)	7 (0.7)	
No	1919 (98.6)	969 (97.9)	950 (99.3)	
Missing data	10	NA	NA	
Ever drinking alcohol				0.006
Yes	640 (32.9)	353 (35.7)	287 (29.9)	
No	1307 (67.1)	635 (64.3)	672 (70.1)	
Missing data	10	NA	NA	
Total CTQ scores	32.0 [28.0, 40.0]	32.0 [28.0, 40.0]	32.0 [28.0, 39.0]	0.756
Total RSES scores	29.0 [26.0, 32.0]	29.0 [26.0, 32.0]	29.0 [25.0, 32.0]	< 0.001
Baseline CESD scores	11.0 [6.0, 18.0]	10.0 [6.0, 16.0]	12.0 [7.0, 19.0]	< 0.001
Baseline SDQ subscale^#^				
SDQ-internalizing	5.0 [3.0, 7.0]	4.0 [3.0, 6.0]	5.0 [3.0, 7.0]	< 0.001
SDQ-externalizing	5.0 [3.0, 7.0]	5.0 [3.0, 7.0]	5.0 [3.0, 7.0]	0.083

SD, standard deviation; HSS, household socioeconomic status; CTQ, Childhood Trauma Questionnaire; RSES, Rosenberg Self-Esteem Scale; CESD, Center for Epidemiology Scale for Depression; SDQ, Strengths and Difficulties Questionnaire.

NA, not applicable or no data available.

^&^The values were presented as median [interquartile range] for non-normal distributions and number (percentage) for categorical variables.

*The chi-square test was used for categorical variables, and the Wilcoxon-Mann-Whitney test for nonnormally distributed continuous variables.

^#^ SDQ-internalizing represents the sum of SDQ emotional symptom subscale and peer relationship problem subscale score; SDQ-externalizing represents the sum of SDQ conduct problems subscale and hyperactivity/inattention subscale score.

### Correlation analysis

The correlations between relevant variables of the hypothesized mediation model are shown in Table 2. The results showed that all the study variables were significantly correlated with each other, either for the overall data or for the sexes separately. For the overall sample, CM (wave 1) was positively associated with internalizing (*r* = 0.284; *p* < 0.001) and externalizing (*r* = 0.353; *p* < 0.001) problems (wave 2), and depressive symptoms (wave 3) (*r* = 0.334; *p* < 0.001). Moreover, self-esteem (wave 1) was significantly negatively correlated with CM (wave 1) (*r* = -0.427; *p* < 0.001), internalizing (*r* = -0.397; *p* < 0.001) and externalizing (*r* = -0.321; *p* < 0.001) problems (wave 2), and depressive symptoms (wave 3) (*r* = -0.388; *p* < 0.001). Meanwhile, internalizing problems (wave 2), externalizing problems (wave 2), and depressive symptoms (wave 3) were positively and significantly correlated with each other (*p* < 0.001). Similar significant correlations between study variables still existed when participants were stratified according to sex. The correlation coefficients ranged from − 0.578 to 0.581 for males and from − 0.640 to 0.689 for females (Table 2). In addition, collinearity was evaluated by the variance inflation factors (VIF). All VIF values are well below the threshold of value 5 (i.e., all VIF < 3.5) which suggests no issues with collinearity [54]. Thus, the constructs in our models do not overlap and can be considered as reliable (Table S1).

**Table 2:** Correlation between CM, self-esteem, internalizing/externalizing problems, and depressive symptoms, overall and stratified by sex

Variables	1	2	3	4	5	6	7	8
Overall								
1. CM (W1)	1							
2. Self-esteem (W1)	-0.427***	1						
3. Internalizing problems (W2)	0.284***	-0.397***	1					
4. Externalizing problems (W2)	0.353***	-0.321***	0.486***	1				
5. Depressive symptoms (W3)	0.334***	-0.388***	0.488***	0.423***	1			
6. Baseline Internalizing problems (W1)	0.367***	-0.487***	0.533***	0.329***	0.397***	1		
7. Baseline externalizing problems (W1)	0.410***	-0.409***	0.325***	0.588***	0.359***	0.417***	1	
8. Baseline depressive symptoms (W1)	0.454***	-0.613***	0.526***	0.411***	0.495***	0.640***	0.514***	1
Stratified by sex								
1. CM (W1)	1	-0.430***	0.308***	0.363***	0.368***	0.415***	0.398***	0.486***
2. Self-esteem (W1)	-0.431***	1	-0.418***	-0.340***	-0.393***	-0.546***	-0.437***	-0.640***
3. Internalizing problems (W2)	0.265***	-0.364***	1	0.479***	0.538***	0.568***	0.347***	0.575***
4. Externalizing problems (W2)	0.343***	-0.319***	0.508***	1	0.447***	0.321***	0.596***	0.399***
5. Depressive symptoms (W3)	0.309***	-0.369***	0.420***	0.422***	1	0.414***	0.379***	0.518***
6. Baseline Internalizing problems (W1)	0.328***	-0.416***	0.482***	0.361***	0.367***	1	0.418***	0.689***
7. Baseline externalizing problems (W1)	0.420***	-0.396***	0.317***	0.574***	0.360***	0.436***	1	0.517***
8. Baseline depressive symptoms (W1)	0.436***	-0.578***	0.458***	0.447***	0.458***	0.581***	0.538***	1

The correlation analysis was performed by using Spearman rank-order correlation. When stratified by sex, the correlations for males are presented below the diagonal, and data for females are present above the diagonal.

W1, wave 1; W2, wave 2; W3, wave 3; CM, childhood maltreatment.

^***^p < 0.001.

### Mediation analysis

**Fig. 2:**
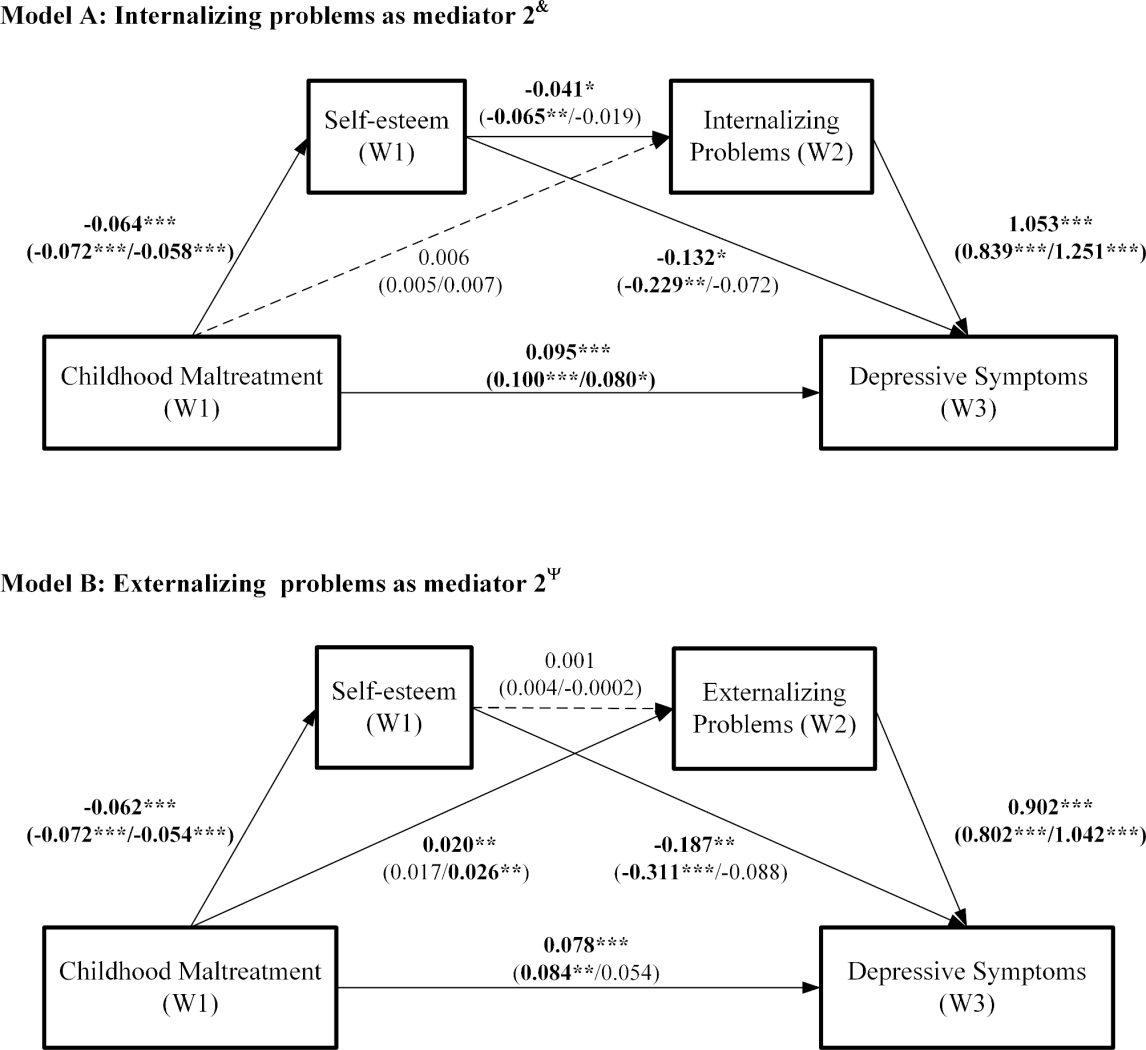
Serial-multiple mediation of self-esteem (wave 1) and different SDQ subscales (wave 2) in the relationship between CM (wave 1) and depressive symptoms (wave 3) Solid arrows indicate significant paths, and dotted arrows indicate insignificant paths The unstandardized path coefficients for the overall sample are presented outside the brackets, and data for males and females are present on the left and right in brackets, respectively. Significant path coefficients have been bolded All mediation models were adjusted for age, household socioeconomic status, living arrangement, classmate relations, relationships with teachers, smoking, drinking, depressive symptoms, internalizing and externalizing problems at wave 1, and sex (overall model only) W1, wave 1; W2, wave 2; W3, wave 3 *p < 0.05, **p < 0.01, ***p < 0.001 ^**&**^ Overall: *R*^2^ = 0.29, *F* = 63.08, *p* < 0.001; male: *R*^2^ = 0.26, *F* = 29.52, *p* < 0.001; female: *R*^2^ = 0.30, *F* = 35.64, *p* < 0.001 ^**Ψ**^ Overall: *R*^2^ = 0.29, *F* = 61.91, *p* < 0.001; male: *R*^2^ = 0.25, *F* = 27.77, *p* < 0.001; female: *R*^2^ = 0.30, *F* = 36.13, *p* < 0.001

The longitudinal serial multiple mediation models were conducted to analyze the mediating role of self-esteem, internalizing problems, and externalizing problems in the relationship between the CM and depressive symptoms. The unstandardized path coefficients of the mediation model for the total sample and by sex are displayed in Fig. 2. Standardized path coefficients are simultaneously presented in Figure S1. The total effect, direct effect, and indirect effect of CM on depressive symptoms are shown in Table 3.

**Table 3:** The direct, indirect, total effects and serial mediation paths in the relationships between CM and depressive symptoms

	Overall				Male				Female		
	*β*	SE	95% CI^*^		*β*	SE	95% CI^*^		*β*	SE	95% CI^*^
**Internalizing problems as mediator 2**											
Total Effect	0.113	0.024	**0.067 to 0.159**		0.124	0.031	**0.064 to 0.185**		0.094	0.036	**0.024 to 0.164**
Direct Effect	0.095	0.023	**0.050 to 0.140**		0.100	0.030	**0.040 to 0.159**		0.080	0.034	**0.013 to 0.146**
Total Indirect Effect	0.018	0.009	**0.0003 to 0.036**		0.025	0.012	**0.002 to 0.049**		0.015	0.015	-0.015 to 0.043
Indirect pathways											
CM→ SE → DS	0.009	0.004	**0.001 to 0.018**		0.017	0.007	**0.004 to 0.033**		0.004	0.005	-0.006 to 0.015
CM →IP → DS	0.006	0.008	−0.010 to 0.022		0.004	0.009	-0.013 to 0.022		0.009	0.014	-0.020 to 0.035
CM → SE →IP → DS	0.003	0.001	**0.001 to 0.006**		0.004	0.002	**0.001 to 0.008**		0.014	0.002	-0.002 to 0.005
**Externalizing problems as mediator 2**											
Total Effect	0.108	0.024	**0.061 to 0.154**		0.120	0.032	**0.058 to 0.183**		0.086	0.036	**0.015 to 0.156**
Direct Effect	0.078	0.023	**0.032 to 0.124**		0.084	0.031	**0.023 to 0.146**		0.054	0.035	-0.015 to 0.123
Total Indirect Effect	0.030	0.008	**0.014 to 0.046**		0.036	0.012	**0.014 to 0.061**		0.032	0.012	**0.008 to 0.056**
Indirect pathways											
CM → SE→ DS	0.012	0.004	**0.004 to 0.021**		0.022	0.008	**0.008 to 0.040**		0.005	0.005	-0.005 to 0.016
CM → EP → DS	0.018	0.007	**0.005 to 0.032**		0.014	0.009	-0.003 to 0.033		0.027	0.012	**0.005 to 0.050**
CM → SE →EP → DS	0.000	0.001	−0.002 to 0.002		-0.0002	0.001	-0.003 to 0.003		0.000	0.001	-0.003 to 0.003

CM, Childhood maltreatment; CI, confidence interval; SE, Self-esteem; DS, Depressive symptoms; IP, Internalizing problems; EP, Externalizing problems.

Note: Adjusted covariates indicating age, household socioeconomic status, living arrangement, classmate relations, relationships with teachers, smoking, drinking, depressive symptoms, internalizing and externalizing problems at baseline, and sex (overall model only). The 95% CI not including 0 indicates statistical significance, given in bold.

^*^95% CI is presented as bias-corrected and accelerated 5,000 bootstrapping.

#### The mediating roles of self-esteem and internalizing problems

As shown in Fig. 2 (model A), all the direct effects were significant in the total sample and males (*p* < 0.05), except for the effect of CM on internalizing problems (path a2, *p* > 0.05). In addition, only the direct effects of CM on depressive symptoms (path c, *β* = 0.080; *p* < 0.05), CM on self-esteem (path a1, *β* = −0.058; *p* < 0.001), and internalizing problems on depressive symptoms (path b2, *β* = 1.251; *p* < 0.001) were significant among female participants. Moreover, the top half of Table 3 shows that, in the total sample and males, self-esteem mediated the association between CM and depressive symptoms (overall, indirect effect = 0.009, 95% CI = 0.001 ~ 0.018; male, indirect effect = 0.017, 95% CI = 0.004 ~ 0.033). Besides, self-esteem and internalizing problems sequentially mediated the path from CM to depressive symptoms (overall, indirect effect = 0.003, 95% CI = 0.001 ~ 0.006; male, indirect effect = 0.004, 95% CI = 0.001 ~ 0.008). However, none of the indirect effects was significant among females, whereas the total and direct effects were significant (total effect = 0.094, 95% CI = 0.024 ~ 0.164; direct effect = 0.080, 95% CI = 0.013 ~ 0.146).

#### The mediating roles of self-esteem and externalizing problems

In Fig. 2 (model B), all direct effects were significant in the total sample (*p* < 0.01), except for the effect of self-esteem on externalizing problems (path a3, *p* > 0.05). Furthermore, the direct effects of CM on self-esteem (path a1) and externalizing problems on depressive symptoms (path b2) were statistically significant in both sexes (*p* < 0.001). Additionally, the direct effects of CM on depressive symptoms (path c, *β* = 0.084; *p* < 0.01), and self-esteem on depressive symptoms (path b1, *β* = −0.311; *p* < 0.001) were only significant in males but not females, while the effect of CM on externalizing problems (path a2, *β* = 0.026; *p* < 0.01) was identified in females rather than males. Furthermore, the lower half of Table 3 shows that self-esteem acted as a partial mediator in the association between CM and depressive symptoms in males (total effect = 0.120, 95% CI = 0.058 ~ 0.183; indirect effect = 0.022, 95% CI = 0.008 ~ 0.040), and the mediating effect accounted for 18.3% of the total effect. For females, externalizing problems played a complete mediating role in the relationship between CM and depressive symptoms (indirect effect = 0.027, 95% CI = 0.005 ~ 0.050).

**Fig. 3:**
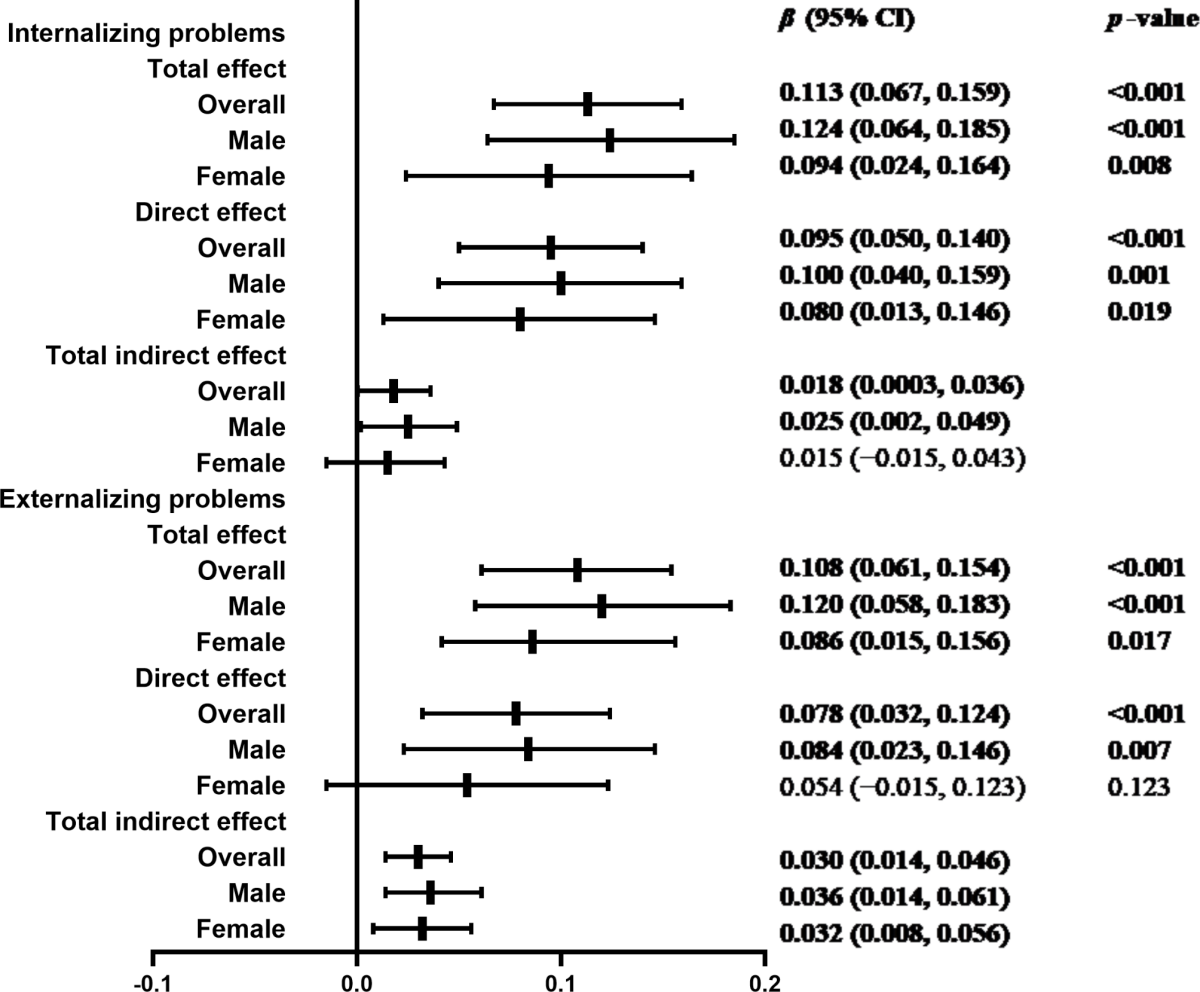
Forest plot for the total, direct, and indirect effects CI, confidence interval. 95% CI is presented as bias-corrected and accelerated 5,000 bootstrapping Note: Adjusted covariates include age, household socioeconomic status, living arrangement, classmate relations, relationships with teachers, smoking, drinking, depressive symptoms, internalizing and externalizing problems at baseline, and sex (overall model only). The 95% CI not including 0 indicates statistical significance, given in bold

Finally, all the above effects are presented in the graphs in Fig. 3. In general, the mediation models accounted for a significant proportion of the variance in the adolescents’ depressive symptoms (Fig. 2-model A/B, overall: *R*^2^ = 0.29/0.29, *p* < 0.001; male: *R*^2^ = 0.25/0.26, *p* < 0.001; female: *R*^2^ = 0.30/0.30, *p* < 0.001).

## Discussion

To our knowledge, this is the first study investigating sex differences in the relationship between CM, self-esteem, internalizing and externalizing problems, and the severity of depressive symptoms among adolescents. The current study revealed that both self-esteem and internalizing/externalizing problems might play an independent or sequential mediating role between CM and depressive symptoms. However, the mediational effect of each variable was different across males and females.

Based on the total study population, our main findings include the following. Firstly, the direct effect measured in our study indicated that experiencing more CM was directly associated with greater levels of depressive symptoms among adolescents, after accounting for baseline levels of depressive symptoms. Recent meta-analyses have found similar results, which reported that childhood abuse and neglect were significantly associated with depressive symptoms in later life, and the odds of developing a mental disorder were more than three times higher for those exposed to multiple forms of maltreatment [9, 10]. According to hopelessness theory, negative life events and negative cognitive styles increase the risk of hopelessness (proximate risk factors), which in turn, causes low self-esteem and depression [12]. Moreover, the stress exposure model posits that early exposure to stressful life events (e.g., CM), contributes to the development of various psychological problems (e.g., depressive symptoms) in later life [55].

Secondly, we explored the role of self-esteem and internalizing problems in the relationship between CM and depressive symptoms. Our major findings are that self-esteem and internalizing problems have serial mediating effects in the relationship between CM and depressive symptoms, and CM is associated with depressive symptoms by decreasing self-esteem levels and increasing internalizing problems, thereby supporting our hypotheses. These findings are in accordance with the previous study conducted among 937 adolescents in Turkey, which reported that self-esteem partially mediated the relationship between maltreatment and internalizing problems [56]. Similar observations were noticed in a study conducted among high school students in China [33]. In this study, CM had significant indirect effects on internalizing behavior problems through self-esteem. Adolescents experiencing CM may have intensely negative individual views of themselves, and they easily conclude that their values and self-worth are low [57, 58]. With distorted self-esteem, adolescents are more prone to have internalizing problems, psychiatric problems, and lower well-being in later life [56, 57].

Thirdly, we explored the role of self-esteem and externalizing problems in the relationship between CM and depressive symptoms. Our study demonstrated that self-esteem mediated the relationship between maltreatment and depressive symptoms, in line with the previous findings [59]. A study in the United States investigated the pathway of different adverse childhood experiences and self-esteem to depressive symptoms among adolescents and found that only the association of child maltreatment with depressive symptoms was mediated by self-esteem [18]. According to attachment theory [13, 14], children who experience CM are likely to develop negative representations of themselves, developing insecure attachment styles, which threaten the development of mental and physical health in the future [60].

Furthermore, our results also indicate that externalizing problems play a mediating role in the relationships between CM and depressive symptoms. Specifically, higher levels of CM are associated with more externalizing problems, which in turn indirectly impacts the development of depressive symptoms. Similarly, Justin et al. also reported that exposure to CM was associated with greater externalizing problems that eventuated in more severe depressive symptoms [30]. It is often assumed that the development of psychiatric disorders is homotypic symptom continuity [61]. That is, individuals with internalizing symptoms in childhood were more likely to have internalizing symptoms (e.g., depression) in later life, while individuals with externalizing symptoms were more likely to have externalizing symptoms (e.g., substance abuse) in later life [61]. However, according to developmental psychopathology concepts, heterotypic continuity, and psychopathological progression, the developmental pattern of psychopathology may exemplify heterotypic symptom continuity and manifest change with increasing age [62]. Therefore, CM may also indirectly affect depressive symptoms through childhood externalizing symptoms.

Interestingly, sex differences were identified in the mediating effects of self-esteem, and internalizing/externalizing problems on the relationship between CM and depressive symptoms. The serial mediating roles of self-esteem and internalizing problems were found only in boys. In females, low self-esteem and internalizing problems did not mediate the relationship between CM and depressive symptoms neither separately nor sequentially. These findings show evidence that sex exerts a significant influence underlying the pathways from exposure of CM to depressive symptoms. This pattern of sex difference is in line with the previous study showing that boys with histories of maltreatment had lower levels of self-esteem, and lower self-esteem was associated with internalizing behavior problems, indicating that self-esteem significantly mediated the relationship between maltreatment and internalizing behavior problems for boys. Additionally, there was no significant mediated effect of self-esteem on internalizing behavior problems for girls [63]. However, this was not consistent with the findings of another study reporting that low self-esteem played a mediating role in the impact of victimization experiences on internalizing problems both for boys and girls [64]. It should be noted that the study measured victimization rather than maltreatment during childhood. Research suggested that self-esteem may have a greater impact among boys than among girls, as boys who have low self-esteem were more likely to suffer from depression than girls who have low self-esteem [65]. Moreover, a 4-year longitudinal comparison group research conducted among male and female youth reported that only girls’ internalizing symptoms served as a mediator between child maltreatment and externalizing behavior [66]. Existing theoretical frameworks may shed light on why these sex differences occur. For example, Chodorow’s theory indicated that females were initially more inclined to adopt an internalizing coping strategy to solve distress, so they suppressed their emotions like anger and aggression for conforming to others’ expectations. However, these maladaptive regulation strategies among girls do not effectively reduce the intensity and frequency of angry or aggression and thus may give rise to adverse behaviors that intensifies over time [67]. Therefore, it can be speculated that in girls, CM may have an effect on externalizing behavior via internalizing problems and consequently on depressive symptoms rather than exerting a direct effect through internalizing problems. As other studies have shown, the potential mediating effect of externalizing behaviors may better explain the links between maltreatment and depressive symptoms in girls [28].

Additionally, our data reveal that self-esteem plays a mediating role between CM and depressive symptoms only in males. This result implies that low self-esteem has a greater impact on adverse health outcomes in males, which is consistent with the previous research [65]. Thus, we should place more emphasis on the long-term health risks due to self-esteem, particularly for boys. Surprisingly, we found that externalizing problems played a complete mediating role in the relationship between CM and depressive symptoms in females. A national birth cohort study prospectively assessed the correlation between maltreatment and subsequent behavior problems, indicating that girls were more likely than boys to develop externalizing behaviors as a result of maltreatment [28]. The externalizing disorder was considered as a particularly salient source of stress for women and increased the risk of depression during adolescence [4]. Therefore, the observed sex differences should be considered in future research when further investigating potential mediators of CM and depression, and the potential effect of mediators on depressive symptoms over time [68].

Taken together, there are several strengths in the present study. First, to our knowledge, it is the first large, three-wave prospective cohort study addressing mediators and sex differences in the association between CM and depressive symptoms. This made it possible to assume temporal precedence with a serial mediation model between variables [69]. Second, we conducted our study on a relatively large sample of school students (n = 1957). Third, we demonstrated the importance of taking sex into account when considering the pathway. The study variables included in this model, such as CM, self-esteem, internalizing and externalizing problems, and depressive symptoms, are likely to be affected by sex and developmental stages. Attention to sex differences can serve to better understand the mechanism of depression and develop effective individualized intervention strategies. Finally, we controlled potential covariates that might be associated with depressive symptoms in the models, making the results more reliable and accurate.

Despite these strengths, this study has several limitations. First, we did not exclude participants with depressive symptoms at baseline, which may bias our results. However, the inclusion of baseline depressive symptoms as a covariate helped to minimize its influence on our results. These findings need to be replicated in a larger sample of non-depressed adolescents. Second, we only examined the effects of overall CM on depressive symptoms, so future studies should explore whether different types of CM produce different effects on depressive symptoms through self-esteem and internalizing or externalizing problems. Third, the effects of CM on depressive symptoms in the current study cannot be precisely explained by serial mediation from low self-esteem to internalizing and externalizing problems. In light of the available literature and the longitudinal data we have collected, assumptions are made concerning the temporal order of the variables in the serial mediation model. Research assessing all the study variables at each time point is warranted to clarify the full causal pathways linking CM and depressive symptoms. Fourth, the measures of CM and self-esteem were obtained concurrently, which may bias the effect of CM on self-esteem. Finally, measures of pubertal timing were not acquired, which has been suggested to influence the onset of depression [70, 71]. Thus, there is a risk of residual confounding by unmeasured covariates. Including pubertal timing as a covariate in future studies is recommended.

## Conclusions

The study herein provides evidence of factors that mediate the association between CM and depressive symptoms in adolescents. Specifically, we reported several mediational factors in the foregoing relationship. Moreover, our analysis identified sex differences in the effect of the identified mediators. For male students, CM exerts an indirect effect on depressive symptoms via self-esteem. Furthermore, the most important finding supports a significant serial mediation chain from low self-esteem to internalizing problems in the association of CM with depressive symptoms in male students. Moreover, CM is associated with depressive symptoms via externalizing problems among female students. Taken together, our results suggest future research assessing the effect of self-esteem and internalizing/externalizing on the association between CM and depressive symptoms to better characterize observed sex differences. Furthermore, the identified sex differences in our study provide supportive evidence that may inform preventative measures and interventions for male and female adolescents based on differential risk for depressive symptoms.

## Electronic supplementary material

Below is the link to the electronic supplementary material.

Supplementary Material 1 Result of the collinearity test of each variable and standardized path coefficients of serial-multiple mediation models

## Data Availability

The datasets used and/or analysed during the current study are available from the corresponding author on reasonable request.

## Abbreviations

CM: Childhood maltreatment
LSAMBR: Longitudinal Study of Adolescents’ Mental and Behavioral Well-being Research
CTQ-SF: Childhood Trauma Questionnaire-Short Form
RSES: Rosenberg Self-Esteem Scale
SDQ: Strengths and Difficulties Questionnaire
CESD: Center for Epidemiologic Studies Depression Scale
HSS: Household socioeconomic status
SD: Standard deviation
CI: Confidence interval.
