# Supplementary material for: Childhood maltreatment and subsequent depressive symptoms: a prospective study of the sequential mediating role of self-esteem and internalizing/externalizing problems
Source: BMC Psychiatry. 2023 Mar 20;23:179. doi: 10.1186/s12888-023-04654-7 (PMC10026476; doi:10.1186/s12888-023-04654-7)
Supplement: Supplementary file 1 — Supplementary Material 1 Result of the collinearity test of each variable and standardized path coefficients of serial-multiple mediation models [file 12888_2023_4654_MOESM1_ESM.docx]

Supplementary Material

| **Table S1.** Result of the collinearity test of each variable. | | | |
| --- | --- | --- | --- |
| Variables | VIF^*^ | | |
|  | Overall | Male | Female |
| Age (year) | 1.10 | 1.16 | 1.07 |
| Sex | 1.08 | - | - |
| Household socioeconomic status | 1.08 | 1.08 | 1.07 |
| Living arrangement | 1.04 | 1.03 | 1.07 |
| Classmate relations | 1.26 | 1.28 | 1.28 |
| Relationship with teachers | 1.24 | 1.27 | 1.22 |
| Ever smoking a cigarette | 1.04 | 1.05 | 1.03 |
| Ever drinking alcohol | 1.12 | 1.15 | 1.11 |
| CM (W1) | 1.44 | 1.37 | 1.54 |
| Self-esteem (W1) | 1.89 | 1.80 | 1.99 |
| Internalizing problems (W2) | 1.96 | 1.92 | 1.99 |
| Externalizing problems (W2) | 2.01 | 2.10 | 2.00 |
| Baseline Internalizing problems (W1) | 2.16 | 1.88 | 2.39 |
| Baseline externalizing problems (W1) | 1.97 | 1.97 | 2.06 |
| Baseline depressive symptoms (W1) | 2.85 | 2.50 | 3.20 |
| * Multiple linear models run with all the independent variable, mediator variables, dependent variable, and covariates.  CM, childhood maltreatment.  Note: Sex was not included as a covariate in sex-stratified models. | | | |


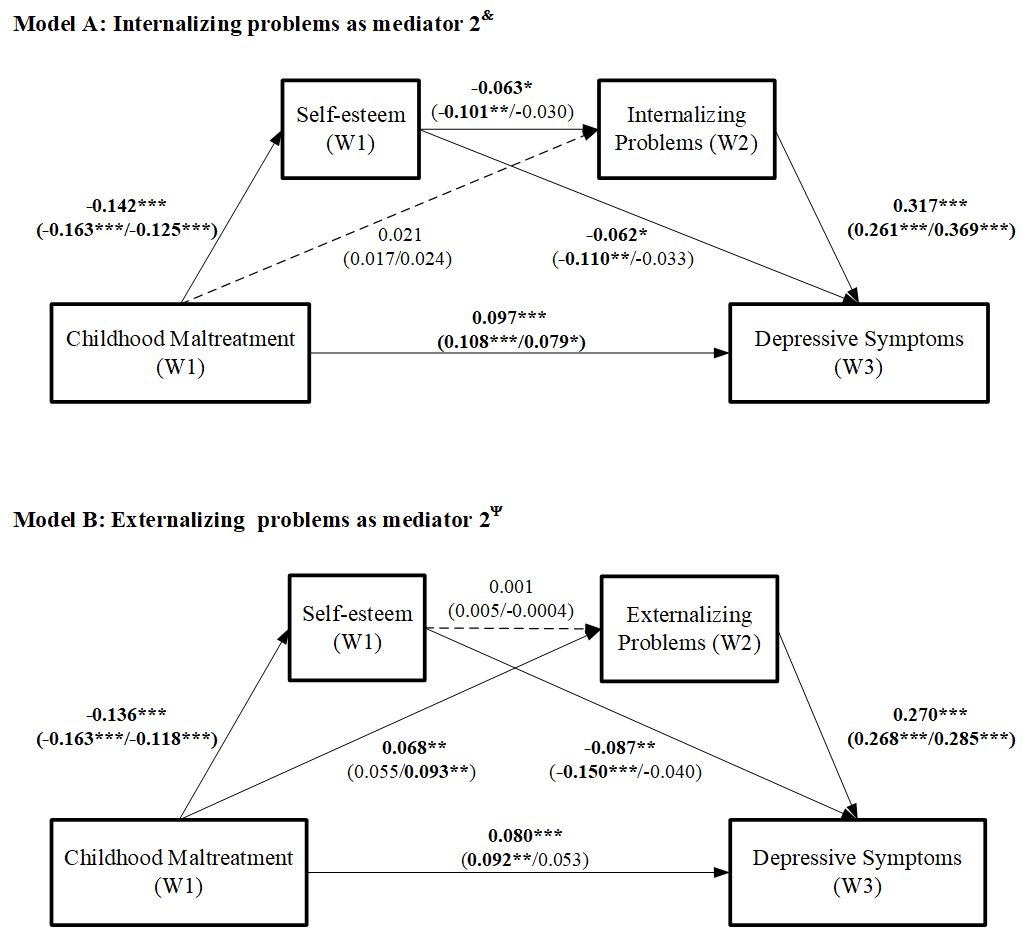


**Figure S1. Serial-multiple mediation of self-esteem (wave 1) and different SDQ subscales (wave 2) in the relationship between CM (wave 1) and depressive symptoms (wave 3).**

Solid arrows indicate significant paths, and dotted arrows indicate insignificant paths.

The standardized path coefficients for the overall sample are presented outside the brackets, and data for males and females are present on the left and right in brackets, respectively. Significant path coefficients have been bolded.

All mediation models were adjusted for age, household socioeconomic status, living arrangement, classmate relations, relationships with teachers, smoking, drinking, depressive symptoms, internalizing and externalizing problems at wave1, and sex (overall model only).

W1, wave 1; W2, wave 2; W3, wave 3.

**p* < 0.05, ***p* < 0.01, ****p* < 0.001.

**^&^** Overall: *R*^2^ = 0.29, *F* = 63.08, *p* < 0.001; male: *R*^2^ = 0.26, *F* = 29.52, *p* < 0.001; female: *R*^2^ = 0.30, *F* = 35.64, *p* < 0.001.

**^Ψ^** Overall: *R*^2^ = 0.29, *F* = 61.91, *p* < 0.001; male: *R*^2^ = 0.25, *F* = 27.77, *p* < 0.001; female: *R*^2^ = 0.30, *F* = 36.13, *p* < 0.001.
